# Investigation of the performance of validated cardiovascular risk scores in a global (UK/US) cohort of young people with childhood-onset systemic lupus erythematosus

**DOI:** 10.1136/lupus-2024-001194

**Published:** 2024-06-13

**Authors:** Coziana Ciurtin, Junjie Peng, Yiming Gao, Misato Niwa, Stacy P Ardoin, Laura Eve Schanberg, Laura Lewandowski, Elizabeth C Jury, George A Robinson

**Affiliations:** 1 Centre for Adolescent Rheumatology Versus Arthritis, Division of Medicine, University College London, London, UK; 2 Centre for Rheumatology Research, Division of Medicine, University College London, London, UK; 3 Faculty of Medical Sciences, University College London, London, UK; 4 Department of Pediatrics, Nationwide Children’s Hospital, Ohio State University, Columbus, Ohio, USA; 5 Duke Clinical Research Institute, Department of Pediatrics, Duke University School of Medicine, Durham, North Carolina, USA; 6 Lupus Genomics and Global Health Disparities Unit, National Institute of Arthritis and Musculoskeletal and Skin Diseases, National Institutes of Health, Bethesda, Maryland, USA

**Keywords:** Atherosclerosis, Lupus Erythematosus, Systemic, Cardiovascular Diseases

Chronic inflammation is one of the recognised drivers of increased cardiovascular disease risk (CVD-risk). Childhood-onset systemic lupus erythematosus (cSLE), characterised by onset before 18 years of age, has a more severe phenotype than adult-onset SLE and is associated with increased CVD-risk starting early in life. As a consequence of this increased risk, 4% of children and young people (CYP) with cSLE recruited to a large UK study experienced at least one CVD-event 2 years postdiagnosis, at a median age of 16 years.^1^ This suggests a critical unmet need for earlier identification of individuals with high CVD-risk for tailored risk management strategies in cSLE.^2^

Carotid intima-media thickness (CIMT) is one of the best predictors of CVD-risk across the life span. However, CVD-risk assessment guidelines recommend the evaluation of various traditional CVD-risk factors and use of CVD-risk scores, rather than vascular scans, for stratified management approaches in general population.^3^

The *A*therosclerosis *P*revention in *P*ediatric *L*upus *E*rythematosus (APPLE) trial, a large interventional clinical trial in cSLE, evaluated the efficacy of atorvastatin in decreasing atherosclerosis progression in CYP aged 10–18 years, using serial CIMT measurements.^4^ Although the trial did not meet the primary endpoint, it provided us with the opportunity to discover a novel serum metabolomic signature associated with a high rate of atherosclerosis progression in cSLE.^5^

As CIMT measurements on vascular scans are not routinely implemented in clinical practice, we explored the comparative performance of four age-appropriate and validated CVD-risk scores in a global cSLE (UK/US) cohort to address a key research priority identified by our PPIE activities.

Demographic data, CVD-risk factors and cSLE characteristics were collected cross-sectionally from two cSLE cohorts: a retrospective University College London (UCL) cohort (N=109, UK) and a prospective APPLE trial cohort (N=121), both stratified based on the metabolomic signature of high CIMT progression identified in the APPLE trial.^5^ The online supplemental figure depicts the CVD-risk stratification of the UCL cohort based on the metabolomic signature identified in the APPLE cohort.

10.1136/lupus-2024-001194.supp1 Supplementary data

QRISK-3, Framingham, Atherosclerotic Cardiovascular Disease scores (validated for age 20–25) and the Pathobiological Determinants of Atherosclerosis in Youth (PDAY) score (validated from age ≥14) were calculated and assessed for performance against cross-validated metabolomic signatures of CIMT progression. We used descriptive statistics, area under the curve, correlation and linear regression analyses.

Mean age/disease duration for the UCL/APPLE cohorts were 26±4.18 years/13.5±4.71 years and 15.60±2.67 years/2.46±2.44 years, respectively (p<0.001). CYP with cSLE in both cohorts have been stratified based on individual CVD-risk score validated cut-offs (table 1). There were no statistically significant differences between the lipid levels or BMI between the different risk categories in either of the two cohorts analysed.

**Table 1:** CVD-risk stratification of two independent cSLE cohorts

**cSLE patient cohorts stratified based on cross-validated metabolomic signatures of CIMT progression^5^**	**The UCL cohort (N=109**) was stratified as: 29.2% low 48.3% moderate and 22.5% high CVD-risk	**The APPLE cohort (N=121**) was stratified as: 28.5% low, 42.4% moderate and 29.1% high CVD-risk
**QRISK-3 score (N**)	**109**	**119**
Very low QRISK-3 risk<5%	92 (84.4%)	98 (82.3%)
Low QRISK-3 risk=5–9.9%	8 (7.3%)	16 (13.4%)
Moderate QRISK-3 risk=10–19.9%	2 (1.8%)	3 (2.5%)
High QRISK-3 risk>20%	7 (6.4%)	2 (1.7%)
**FRS score (N**)	**80**	**119**
Very low FRS<5%	80 (100%)	119 (100%)
Low FRS=5–9.9%	0	0
Moderate FRS=10–19.9%	0	0
High FRS>20%	0	0
**ASCVD score (N**)	**62**	**80**
Low ASCVD risk<5%	60 (96.8%)	73 (91.1%)
Moderate ASCVD risk=5–7.4%	1 (1.6%)	3 (3.8%)
High ASCVD risk=7.5–20%	1 (1.6%)	4 (5.0%)
**PDAY score (N**)	**78**	**116**
Very low PDAY score<2 points	6 (7.7%)	84 (72.4%)
Low PDAY score=2–5 points	21 (26.9%)	22 (19.0%)
Moderate PDAY score=6–10 points	17 (21.8%)	6 (5.2%)
High PDAY score>10 points	34 (43.6%)	4 (3.5%)

APPLE, Atherosclerosis Prevention in Pediatric Lupus Erythematosus; ASCVD, Atherosclerotic Cardiovascular Disease; CIMT, carotid intima media thickness; cSLE, childhood-onset systemic lupus erythematosus; CVD, cardiovascular disease; FRS, Framingham Risk Score; N, number of children and young people with cSLE with complete data available to calculate various CVD-risk scores; PDAY, Pathobiological Determinants of Atherosclerosis in Youth; UCL, University College London.

All scores had very low performance against CVD-risk metabolomic stratification. The PDAY-score performed best, with 67% *specificity,* but only 50% *sensitivity,* in correctly classifying CYP with high CVD-risk, but only in the UCL cohort, which was older. PDAY correlated with individual’s age, disease duration, median SLEDAI and Paediatric SLICC Damage Index (Ped-SDI) scores (r=0.78, 0.48, 0.28 and 0.3, respectively, p<0.05) in the UCL cohort.

Linear regression analysis found that age/disease activity was the strongest determinant of PDAY-score (1-year increase in age/one point increase in median SLEDAI-2K score over the disease course were associated with 1.13/0.41 points increase in PDAY-score, respectively, when corrected for sex/disease duration/damage/lipid levels/steroids).

Overall disease activity and age were the strongest predictors of PDAY-score, but only in the UCL older cSLE cohort. As age is one of the most important drivers of CVD-risk, to ensure that both cohorts were stratified in a reliable way, we have used a cross-validated metabolomic signature, as we previous demonstrated that these signatures can be validated across age in SLE.^6^

CVD-risk scores, even if validated for ages ≥14, do not adequately capture CVD-risk in adolescents with cSLE (APPLE trial cohort). PDAY-score performed moderately well for young adults only (UCL cohort), highlighting the need for better CVD-risk stratification tools, especially for CYP with cSLE, as we argued before.^2^

Although very few CYP with cSLE have been stratified as having high CVD-risk by any of the CVD-risk scores used in the APPLE cohort at baseline, we also assessed stratification performance of these scores at the end of the APPLE trial (36 months) in the placebo versus statin arm. As the metabolomic signature of atherosclerosis progression we discovered did not predict the CIMT progression in response to statin, we used the rates of CIMT progression over 36 months in each arm^5^ for stratification. As expected, all CVD-risk scores underperformed at the end of the trial as they did at baseline and failed to identify individuals with high CIMT progression rates, irrespective of the treatment arm allocation (online supplemental table). In summary, this study highlights the lack of adequate CVD-risk scores that can be easily implemented in routine clinical practice in cSLE and need for age and disease appropriate CVD-risk stratification tools. Future research and implementation of more sensitive biomarkers (such as metabolomic signatures) may be warranted for optimised CVD-risk identification and management in cSLE.

## Data Availability

Data are available in a public, open access repository. The APPLE clinical trial study protocol and results are publicly available—Use of atorvastatin in systemic lupus erythematosus in children and adolescents—PubMed (nih.gov). Preliminary analyses of this study are also available at SSRN: https://ssrn.com/abstract=4336159 or http://dx.doi.org/10.2139/ssrn.4336159. The study has been reported according to the Consolidated Standards of Reporting Trials (CONSORT) reporting guidelines (PMID: 22031171). Data used for all the complementary analyses included in this manuscript and the analytic codes are available on request.
