# Supplementary material for: Investigation of the performance of validated cardiovascular risk scores in a global (UK/US) cohort of young people with childhood-onset systemic lupus erythematosus
Source: Lupus Sci Med. 2024 Jun 13;11(1):e001194. doi: 10.1136/lupus-2024-001194 (PMC11177662; doi:10.1136/lupus-2024-001194)

Supplementary materials

Investigation of the performance of validated cardiovascular risk scores in a global (UK/US) cohort of young people with childhood-onset systemic lupus erythematosus.

Authors:

Coziana Ciurtin[1,2*], Junjie Peng[1,2], Yiming Gao[3], Misato Niwa[3], Stacy P. Ardoin[4], Laura E. Schanberg[5], Laura Lewandowski[6], Elizabeth C Jury[2], George A Robinson[1,2]

Supplementary Table. CVD-risk stratification of the placebo vs. statin arms of the APPLE cohort at the end of the trial (36 months). The CVD-risk stratification was compared against the CIMT progression over 36 months, as published before (5).

Legend: CIMT= carotid intima media thickness; CVD= cardiovascular disease; N= number of children and young people with cSLE with complete data available to calculate various CVD-risk scores.

**cSLE patient cohorts stratified based on CIMT progression rates over 36 months in the placebo (high vs. low) and statin (high vs. moderate vs. low) arms (6)**	**The placebo group (N=60) at 36M**<br>was stratified as:<br>41.7% low<br>and<br>58.3% high CVD-risk	**The statin group (N=61) at 36M**<br>was stratified as:<br>24.6% low,<br>39.3% moderate, and<br>36.1% high CVD-risk
**QRISK-3 score (N)**	**51**	**55**
Very low QRISK-3 risk <5%	44 (86.3%)	46 (83.6%)
Low QRISK-3 risk = 5-9.9%	5 (9.8%)	7 (12.7%)
Moderate QRISK-3 risk = 10-19.9%	1 (2.0%)	1 (1.8%)
High QRISK-3 risk >20%	1 (2.0%)	1 (1.8%)
**FRS score (N)**	**51**	**55**
Very low FRS <5%	51 (100%)	55 (100%)
Low FRS = 5-9.9%	0	0
Moderate FRS =10-19.9%	0	0
High FRS >20%	0	0
**ASCVD score (N)**	**32**	**23**
Low ASCVD risk <5%	31 (96.9%)	21 (91.3%)
Moderate ASCVD risk = 5-7.4%	0 (0%)	0 (0%)
High ASCVD risk = 7.5-20%	1 (3.1%)	2 (8.7%)

**PDAY score (N)**	**51**	**55**
Very low PDAY score <2 points	32 (62.7%)	29 (52.7%)
Low PDAY score = 2-5 points	10 (19.6%)	15 (27.3%)
Moderate PDAY score = 6-10 points	7 (13.7%)	9 (16.4%)
High PDAY score>10 points	2 (3.9%)	2 (3.6%)

Supplementary Figure:

UCL cohort stratification based on 6-metabolite signature of high CIMT progression in the APPLE trial (5).

Legend: Total – CE = total cholesteryls esters; Total-C = total cholesterol; M-LDL-FC = free cholesterol in medium low-density lipoproteins (LDL); S-LDL-PL = phospholipids in small LDL; S-LDL-C = cholesterol in small LDL; S-LDL-L = total lipids in small LDL

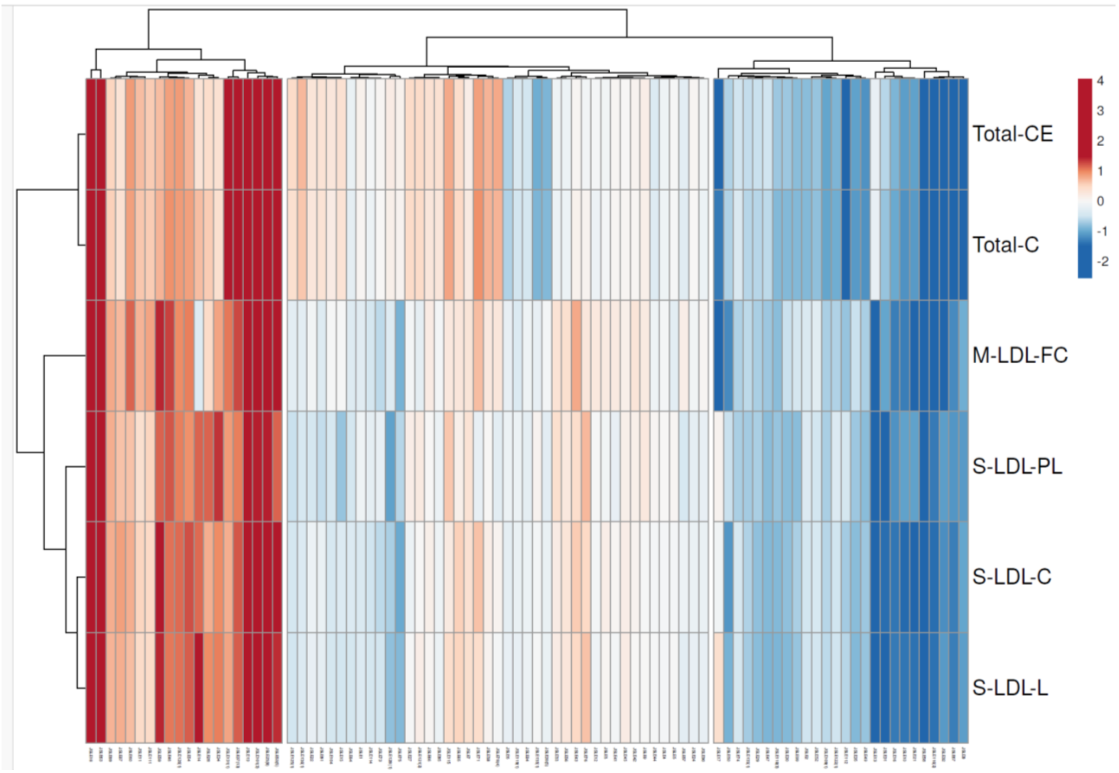

Supplement: Supplementary data [file lupus-2024-001194supp001.pdf]
